# Leveraging large language models for data analysis automation

**DOI:** 10.1371/journal.pone.0317084

**Published:** 2025-02-21

**Authors:** Jacqueline A. Jansen, Artür Manukyan, Nour Al Khoury, Altuna Akalin

**Affiliations:** 1 Max Delbrück Center for Molecular Medicine in the Helmholtz Association (MDC), Berlin, Germany; 2 Bioinformatics and Omics Data Science Platform, Berlin, Germany; 3 University of Potsdam, Potsdam, Brandenburg, Germany; 4 Free University of Berlin, Berlin, Germany; Fundación Universitaria del Área Andina, COLOMBIA

## Abstract

Data analysis is constrained by a shortage of skilled experts, particularly in biology, where detailed data analysis and subsequent interpretation is vital for understanding complex biological processes and developing new treatments and diagnostics. One possible solution to this shortage in experts would be making use of Large Language Models (LLMs) for generating data analysis pipelines. However, although LLMs have shown great potential when used for code generation tasks, questions regarding the accuracy of LLMs when prompted with domain expert questions such as omics related data analysis questions, remain unanswered. To address this, we developed *mergen*, an R package that leverages LLMs for data analysis code generation and execution. We evaluated the performance of this data analysis system using various data analysis tasks for genomics. Our primary goal is to enable researchers to conduct data analysis by simply describing their objectives and the desired analyses for specific datasets through clear text. Our approach improves code generation via specialized prompt engineering and error feedback mechanisms. In addition, our system can execute the data analysis workflows prescribed by the LLM providing the results of the data analysis workflow for human review. Our evaluation of this system reveals that while LLMs effectively generate code for some data analysis tasks, challenges remain in executable code generation, especially for complex data analysis tasks. The best performance was seen with the self-correction mechanism, in which self-correct was able to increase the percentage of executable code when compared to the simple strategy by 22.5% for tasks of complexity 2. For tasks for complexity 3, 4 and 5, this increase was 52.5%, 27.5% and 15% respectively. Using a chi-squared test, it was shown that significant differences could be found using the different prompting strategies. Our study contributes to a better understanding of LLM capabilities and limitations, providing software infrastructure and practical insights for their effective integration into data analysis workflows.

## Introduction

Data analysis often faces bottlenecks due to the scarcity of experts in the field, as the specialized skills required for data manipulation and interpretation are not widely available [1]. This shortage can lead to delays and challenges in extracting actionable insights from data, hindering decision-making and innovation in various sectors. In biology, the scarcity of data analysis experts significantly impedes research progress and discovery, as the interpretation of biological data is crucial for understanding processes such as disease mechanisms, and developing new treatments [2]. Being able to develop well-constructed and accurate data analysis pipelines is the major practical bottleneck for these procedures.

The use of “Large Language Models” (LLMs) for code generation tasks has shown promising results, with generated code snippets matching the expected output for the given task in many cases [3–5]. However, the generated code may not always be optimal, and manual refinement is often required. Investigation into the accuracy of LLMs in solving certain tasks has been an emerging field of interest, with some even reporting an accuracy of 97.3% when using LLMs for introductory bioinformatics tasks [6]. In this study, it was shown that when prompting ChatGPT with introductory bioinformatics coding tasks, 75.5% percent of the tasks were solved upon the first try. Within 7 or fewer attempts, ChatGPT solved 97.3% of the exercises after natural language feedback, highlighting the potential of harnessing LLMs for bioinformatics code generation tasks. However, when using LLMs for the generation of code for more complex data analysis tasks, LLMs might not always provide a reliable or executable answer, and further processing steps might be required [7]. While LLMs have demonstrated exceptional performance across a range of benchmarks, assessing their effectiveness in expert applications poses challenges [8–10]. The absence of a standardized evaluation method is a problem, particularly considering that open-source benchmarks often fall short in measuring performance in specific expert domain settings. These benchmarks are typically crafted to assess general language capabilities rather than certain demands of domain-specific applications such as writing code for omics related data analysis pipelines. Efforts have been made to craft benchmarks for more domain-expert settings, leading to the development of benchmarks such as BioInfo-Bench [9]. Bioinfo-Bench is an evaluation suite, which is constructed of 200 questions covering multiple-choice, sequence verification, and analytical problem-solving tasks. However, no tasks involving writing code are assessed. Consequently, essential questions regarding the accuracy of LLMs when prompted with domain expert questions involving the generation of code, still remain unanswered. Nonetheless, Bioinformatics communities have been actively working to address challenges regarding the usage of LLMs for expert-domain settings. Tools such as bioMANIA, DrBioRight, Bio-Informatics Agent (BIA) and Auto Bioinformatics Analysis (AutoBA) have been developed to leverage LLMs for assisting with a range of different bioinformatics tasks [11–14]. Providing a chat-like interface, DrBioRight is a tool which is designed to be able to conduct somewhat standard analyses of omics data using natural language [12]. Although useful, DrBioRight relies on predefined analytic tasks, rendering the tool incompatible with queries which require the usage of something other than the predefined analytic tasks in the database. bioMANIA makes use of a ChatBot generation pipeline, which extracts information about well-documented Python tools so it can use these when responding to the user query [11]. Although this tool shows great potential, its effectiveness was only tested on single-cell data, and might not translate well when user files are not in a standardized format. BIA is another single-cell data chatbot, which can make use of both public and private databases in performing single-cell analysis [13]. Making use of a RAG database, the query is used to find the most comparable bioinformatics tool use cases reference. Using these references, the LLM is then asked to adapt the code based on the most comparable reference, along with information about variables and packages loading in the environment. Admittingly another great tool, generalization beyond single-cell RNA sequencing tasks was not tested. A tool which seems to be more generalizable across various omics related tasks is AutoBA, including whole genome sequencing, RNA sequencing, single-cell RNA-seq, ChIP-seq, and spatial transcriptomics [14]. A useful tool for conventional bioinformatics analysis tasks, its drawback is that usage of non-standardized file formats might render the tool unusable, since non-standard file formats were not assessed. Other tools such as AutoML and AutoProteinEngine aim to use LLMs for automated machine learning purposes [15, 16]. While all these tools are useful for specific bioinformatics tasks, they are often limited by their narrow focus and/or reliance on predefined formats. For more flexible and comprehensive assistance across a wider variety of coding tasks in the omics field, a foundation LLM together with prompt engineering may prove to be a more robust solution. Such a model can offer the adaptability needed to handle diverse user queries in omics research, which often span numerous analysis types, datasets, and file formats. This is beneficial, since the sort of task is not limited to a certain scope.

In this study therefore, we set out to analyze the efficacy of GPT-3.5-turbo and GPT-4 in writing R-based code when prompted with omics related data analysis questions of all different sorts, and using non-standard file formats. To get an understanding of how the executability of generated code for specific data analysis questions decreases when task complexity is increased, we investigated the executability of LLM generated code when prompted with bioinformatics omics analysis related tasks of various complexities. We hypothesized that by making use of various prompting strategies and self-correction steps, LLM accuracy might be increased.

To enhance the efficacy of LLM in code generation, prompt engineering techniques such as “Act As” (role prompting) and “Chain of Thought” (CoT) could be used. The “Act As” approach in prompt engineering involves instructing the LLM to emulate the reasoning or problem-solving style of an expert or a specific professional [17]. For instance, by prompting the LLM to “Act as a seasoned data analyst and R programmer,” the model is guided to consider the nuances and methodologies typically employed by professionals in that field. This approach could lead to more practical, efficient code generation, as it aligns the model’s output with professional expertise. On the other hand, the “Chain of Thought” (CoT) method breaks down the problem-solving process into a series of logical steps, similar to how a human expert might approach a complex task [18]. By prompting the model to explicitly detail each step in the code generation process, CoT can significantly enhance the output. This approach also makes the model’s reasoning process transparent, enabling users, especially those without extensive programming knowledge, to understand and modify the generated code more effectively. Incorporating these prompt engineering techniques into LLMs for code generation tasks has the potential to produce more reliable and executable code.

**Fig 1:**
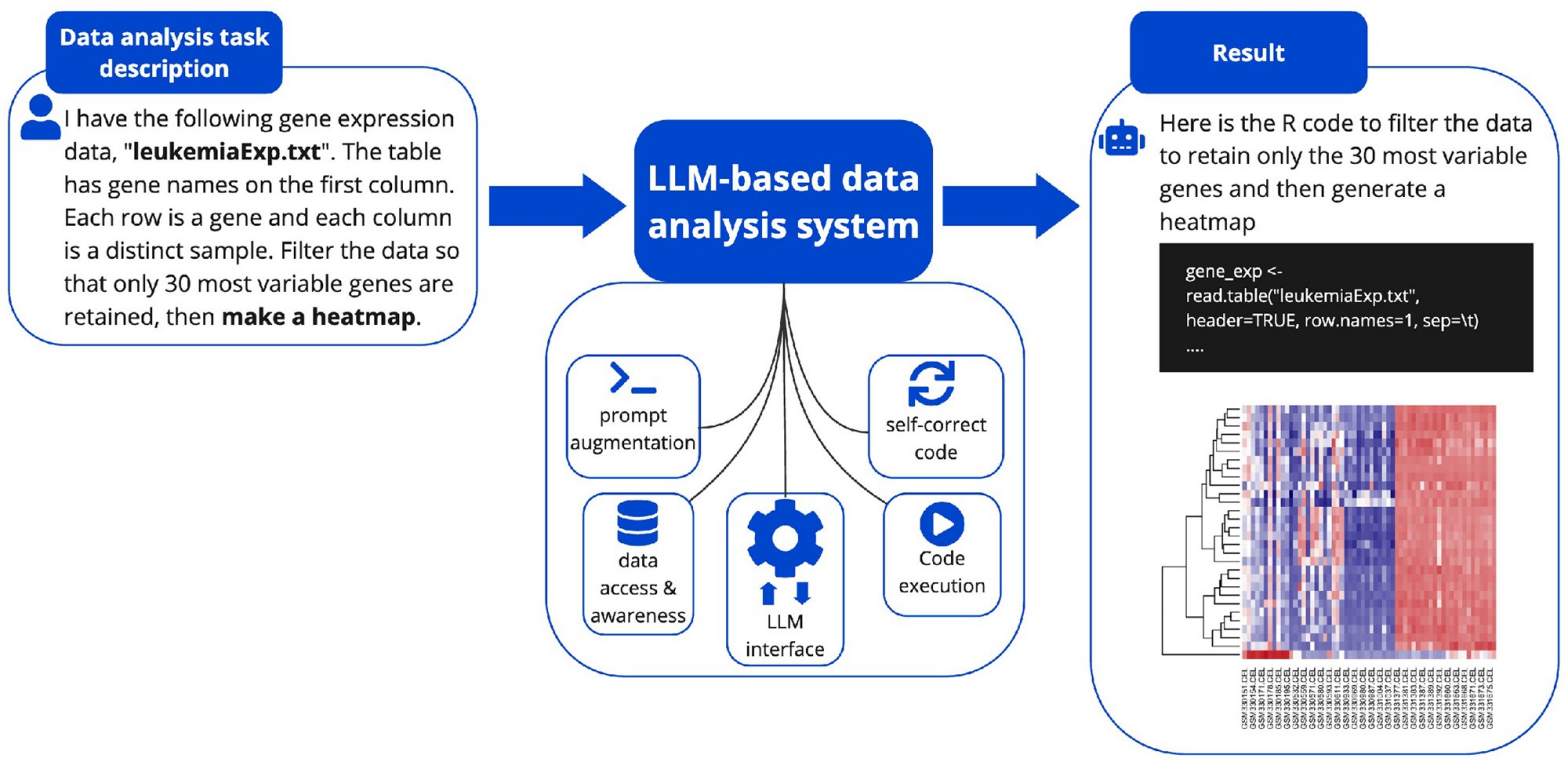
The summary of the LLM-based data analysis system and its features. Given a task description our system can generate and execute the code to carry out such analysis. It interacts and uses the user data that is mentioned in the task. It can correct code execution errors using the implemented self-correction mechanism.

To investigate this, we developed the R package mergen, which features a user-friendly interface for interacting with LLMs, and aids in the help of generating executable code by making use of self-correcting steps (Capabilities and workflow summarized in Fig 1). Mergen enhances the capabilities of LLMs through advanced prompt engineering techniques, the integration of external data files within prompts, and the implementation of error feedback systems. Additionally, it supports real-time execution of generated code, and automated dependency resolution providing a robust platform for model LLM and code development. We present a comprehensive analysis of the code snippets generated by LLM models using various prompt engineering techniques. We examine the performance on a variety of prompts related to omics data analysis, including data preprocessing, exploratory data analysis, data visualization, statistical inference and machine learning applications. Our findings indicate that although LLMs are effective in generating code for simple queries, they still have limitations when it comes to generating executable code for complex tasks.

Our study contributes to the growing body of literature on LLM capabilities and limitations. It provides useful insights and recipes for researchers and practitioners working on data analysis and want to incorporate LLMs into their workflows to increase their productivity especially in genomics and bioinformatics.

## Data description: Tasks examined and evaluation criteria

We examined tasks with different complexity in the domain of bioinformatics. Most tasks are applications of machine learning, statistics, visualization techniques, and data-wrangling methods. We have classified the tasks into different complexity values depending on the steps needed to carry out the tasks. Broadly speaking, these tasks share common components, such as the manipulation and analysis of data stored in files. Each task has one or more of the following components:

- Read data from file(s)
- Data wrangling (filtering, transposing, etc.)
- Visualization
- Machine learning or statistics applications
- Handling more than one dataset

The more of these components a task possesses, the more complex it is ranked. For example, a task that just needs data reading from a file will have complexity 1 and a task that has all of these 5 components above will have complexity 5 (see example tasks in Table 1). Since we expected more complex tasks to have longer response lengths, this was used as a proxy metric for task complexity as well. Below it is shown that this generally also seems to be the case. Tasks used for evaluation are available at our manuscript repository https://github.com/BIMSBbioinfo/mergen-manuscript and can also be found in S1 Table.

**Table 1:** Example prompts, their task-related features, and their assigned complexity values.

Prompt	Task features	Task complexity
I have a tab separated file called “subjects.txt”. Read it and tell me how many rows and columns it has.	Reading a file	1
I have the following gene expression data, “leukemiaExp.txt”. The file is tab separated. The table has gene names on the first column. Each row is a gene and each column is a distinct sample. Filter genes based on their variability so we retain the most variable top 1000 genes. Based on these variable genes cluster the samples, and extract cluster specific genes for each cluster.	Reading a file, Data wrangling Visualization, Machine learning/Statistics application	4

In general, the tasks we have examined are typical bioinformatics data analysis tasks such as clustering and/or application of machine learning methods. Common components across these tasks include data extraction from structured formats, data cleansing to ensure quality and consistency, and data transformation for analysis. LLMs are capable of generating code that is designed to be used for the data stored in files, often in formats like CSV, JSON or tabular text files. In our specific case, the data for tasks are mostly stored in tab-delimited text files or Excel sheets.

For this study, the successful execution of the code is the primary criterion for evaluating the responses that the LLM provides. For all experiments, all 20 prompts were evaluated 10 individual times to account for variability in the generative capabilities of LLMs and to get a more complete picture of their performance.

## Results

We have assessed the capabilities of LLMs and our software package in data analysis tasks. Our evaluation highlights their strengths whilst also identifying areas of improvement, offering insight into practical applications of LLM generated code in data analysis workflows.

### Task complexity reduces code executability

**Fig 2:**
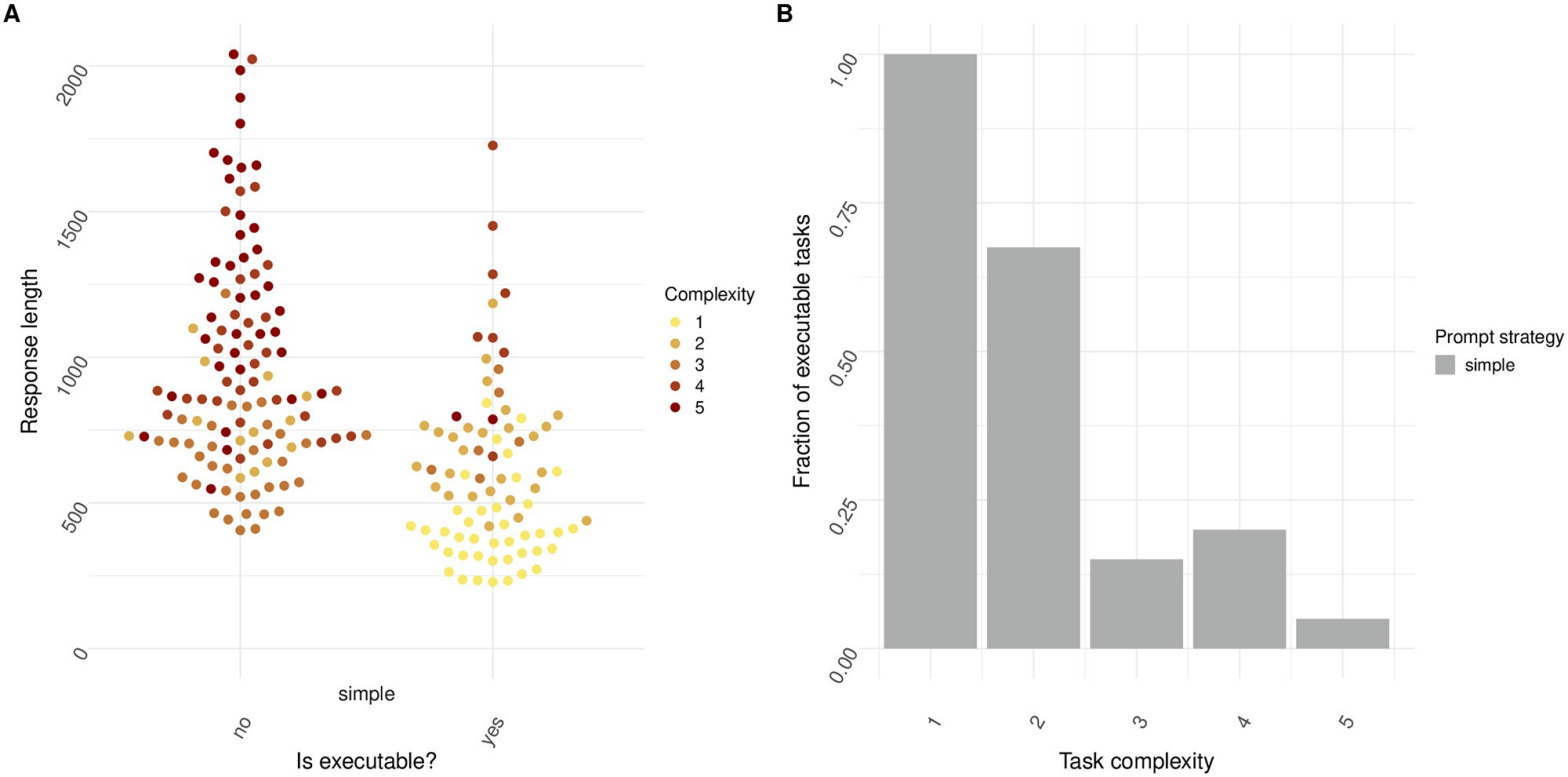
Error rate and fraction of executable tasks is dependent on task complexity and response length for a simple prompt strategy. (A) Executability plotted against response length for tasks of varying complexity. Yes indicates that response code was executable, whereas no indicates response code was not executable. The prompt strategy was set to ‘simple’ (N = 20 individual prompts over n = 10 cycles). (B) Fraction of executable tasks plotted for tasks of increasing complexity. Prompt strategy was set to ‘simple’ (N = 20 individual prompts over n = 10 cycles).

To determine a baseline to see as to what degree LLMs were able to solve tasks of varying complexity, first the simple prompting strategy was investigated when deploying GPT-3.5-turbo. As shown in Fig 2, code executability drastically decreases as task complexity is enhanced. Responses that generated non-executable code were generally longer than responses that resulted in executable code (Fig 2A). Responses that generated executable code were on average 607 characters long, whereas responses that generated inexecutable code were on average 963 characters long. Moreover, tasks that were more complex were more likely to generate longer responses, suggesting that as task complexity increases, response length increases as well, and the fraction of executable code declines. Complex tasks went beyond the simpler tasks such as basic data reading. These results suggests that as tasks grow more complex, general LLMs are not be able to reliably generate a responses which results in executable code.

### Effects of engineered prompts

To explore the effects of prompt engineering on LLMs, prompt engineering strategies “Act as” and “Chain of Thought” (CoT) were employed. In “Act as”, the model is prompted to emulate the thinking process or problem-solving strategy of an expert or a specific role, like a data scientist or a bioinformatician. “Chain of Thought” (CoT) involves constructing prompts that encourage the model to break down a problem into smaller, more manageable steps. This step-by-step approach often aids in clarifying the thought process, making it easier for the model to navigate complex tasks and generate more accurate and executable code.

**Fig 3:**
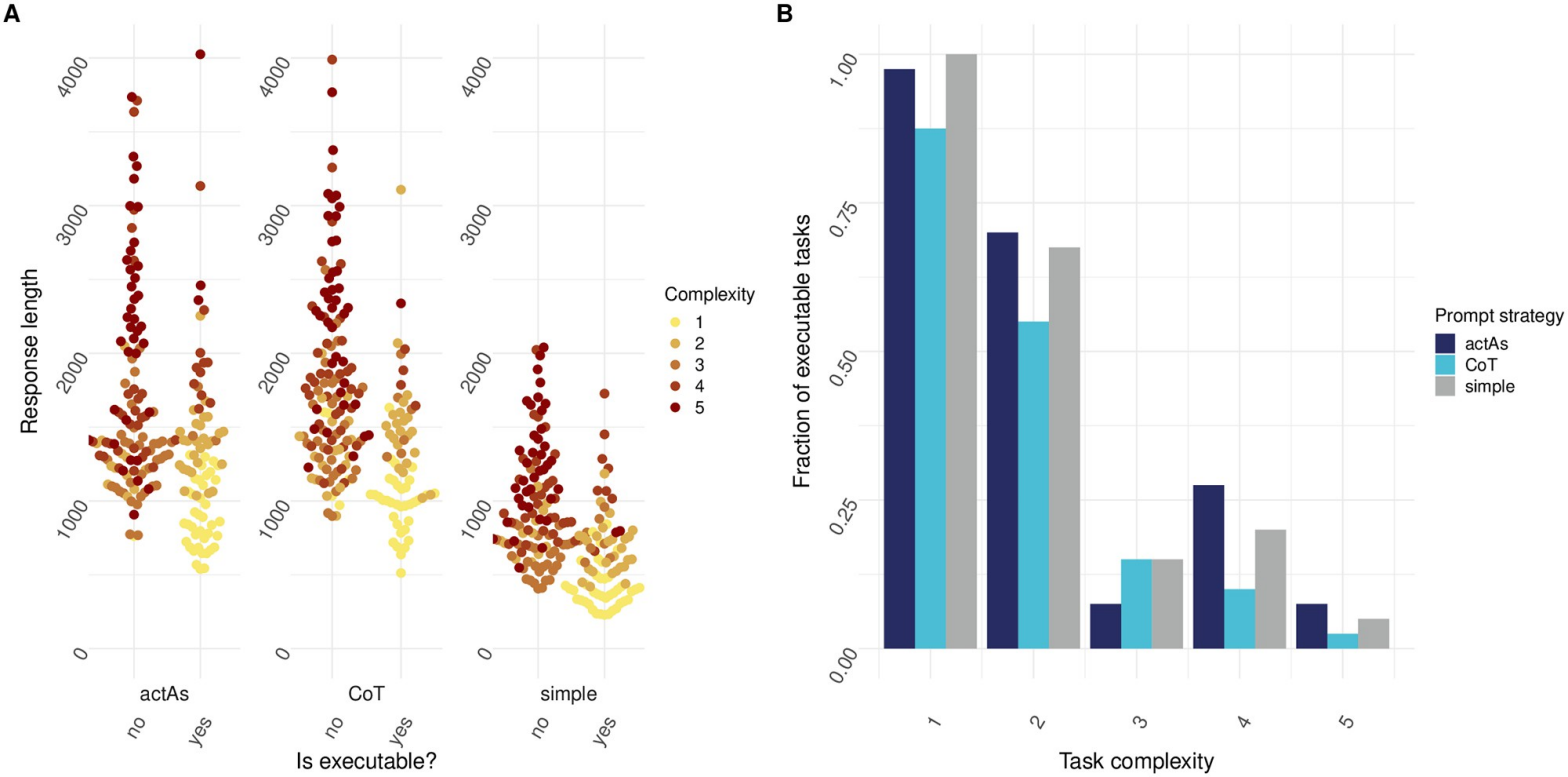
ActAs and CoT prompt strategies do not result in decreased error rate for tasks with increasing complexity. A) Executability plotted against response length for tasks of varying complexity. Yes indicates that response code was executable, whereas no indicates response code was not executable. Prompt strategy was set to “simple”, “CoT” or “ActAs” (N = 20 individual prompts over n = 10 cycles). (B) Fraction of executable tasks plotted for tasks of increasing complexity. Prompt strategy was set to “simple”, “CoT” or “ActAs” (N = 20 individual prompts over n = 10 cycles).

Contrary to our initial hypothesis, our results indicate that more complex prompt engineering techniques do not necessarily lead to a marked improvement in the quality of the generated code. As shown in Fig 3, making use of “CoT” and “ActAs” prompting strategies deploying GPT-3.5-turbo did not increase code executability for more complex tasks, but for the “ActAs” strategy was rather comparable to the simple prompting strategy. When looking at “CoT”, its performance was worse for tasks of almost all different task complexities. When looking at the response length for prompting strategy “ActAs”, responses that generated executable code were on average 1302 characters long, whereas responses that generated inexecutable code were on average 1690 characters long. For “CoT”, responses resulting in executable code were on average 1279 characters long whereas non-executable answers were on average 1811 characters long. This observation suggests that while prompt engineering can steer the model in the desired direction, the inherent capabilities of the model and the nature of the task itself play more significant roles in determining the outcome. It is however important to note that although code executability did not increase when using these prompt engineering steps, overall task adequateness was not assessed.

### Data file content inclusion improves responses

**Fig 4:**
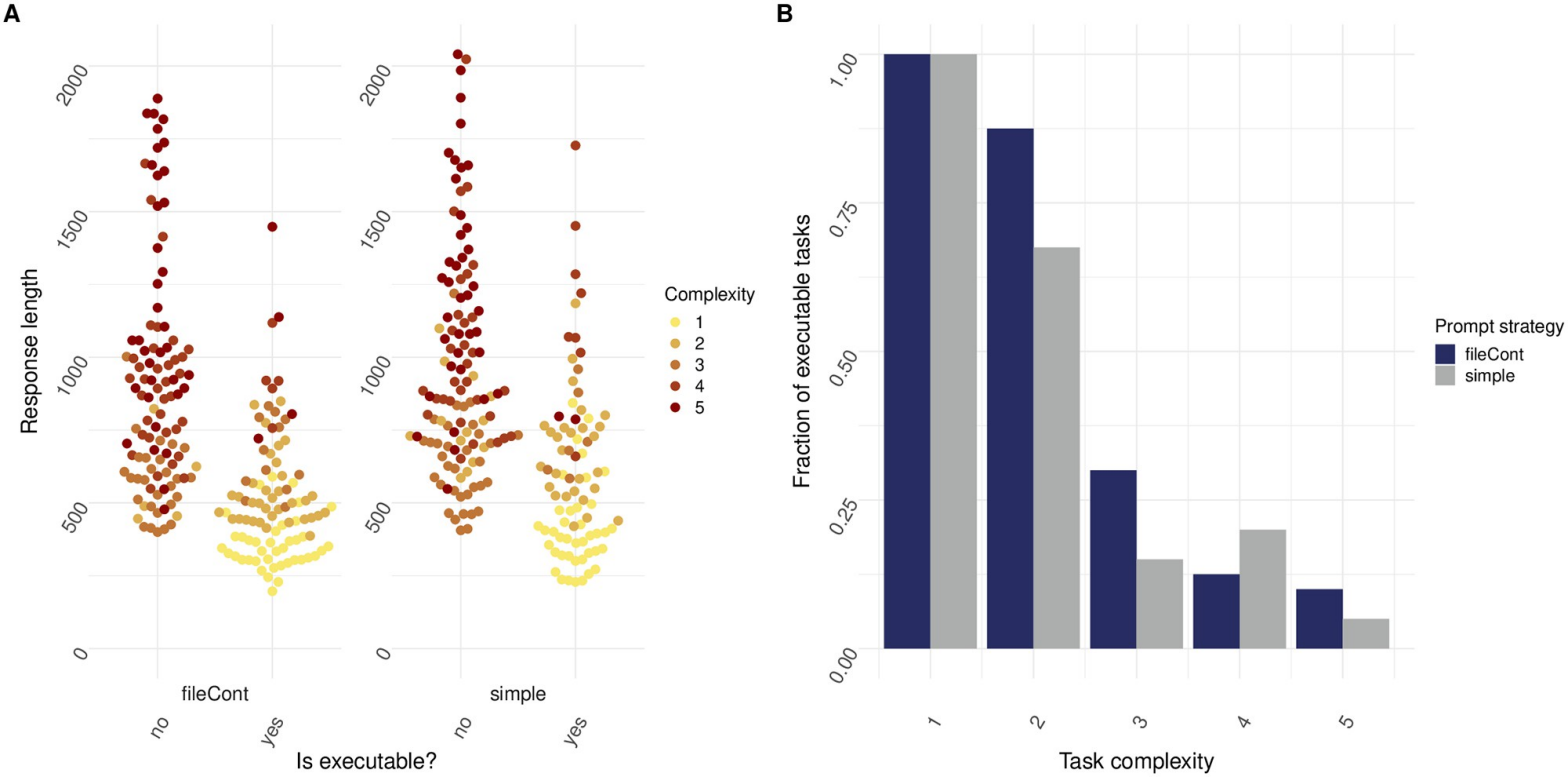
fileCont prompt strategy results in decreased error rate for some tasks with increasing complexity. (A) Executability plotted against response length for tasks of varying complexity. Yes indicates that response code was executable, whereas no indicates response code was not executable. Prompt strategy was set to “simple” or “fileCont” (N = 20 individual prompts over n = 10 cycles). (B) Fraction of executable tasks plotted for tasks of increasing complexity. Prompt strategy was set to “simple” or “fileCont” (N = 20 individual prompts over n = 10 cycles).

As many of the tasks contain a data wrangling step, we set out to see if file content inclusion might increase LLM accuracy. We investigated whether adding the first two lines of files mentioned in the prompts resulted in increased code executability. We hypothesized that this should enable better code since dataset descriptions in the prompt may not be enough for the LLM to generate task code which is executable. The dataset header inclusion is achieved by reading the data file contents and appending the first few lines at the end of the prompt. In some cases, this improved the executability of the code generated by LLMs dramatically, showing for instance an increase in code executability of 20% for tasks of complexity 2, and 15% for tasks of complexity 3. However, for tasks of complexity 4 or higher, adding file content to the prompt seemed to increase the error rate (Fig 4).

### Effect of self-correction mechanism for code generation

**Fig 5:**
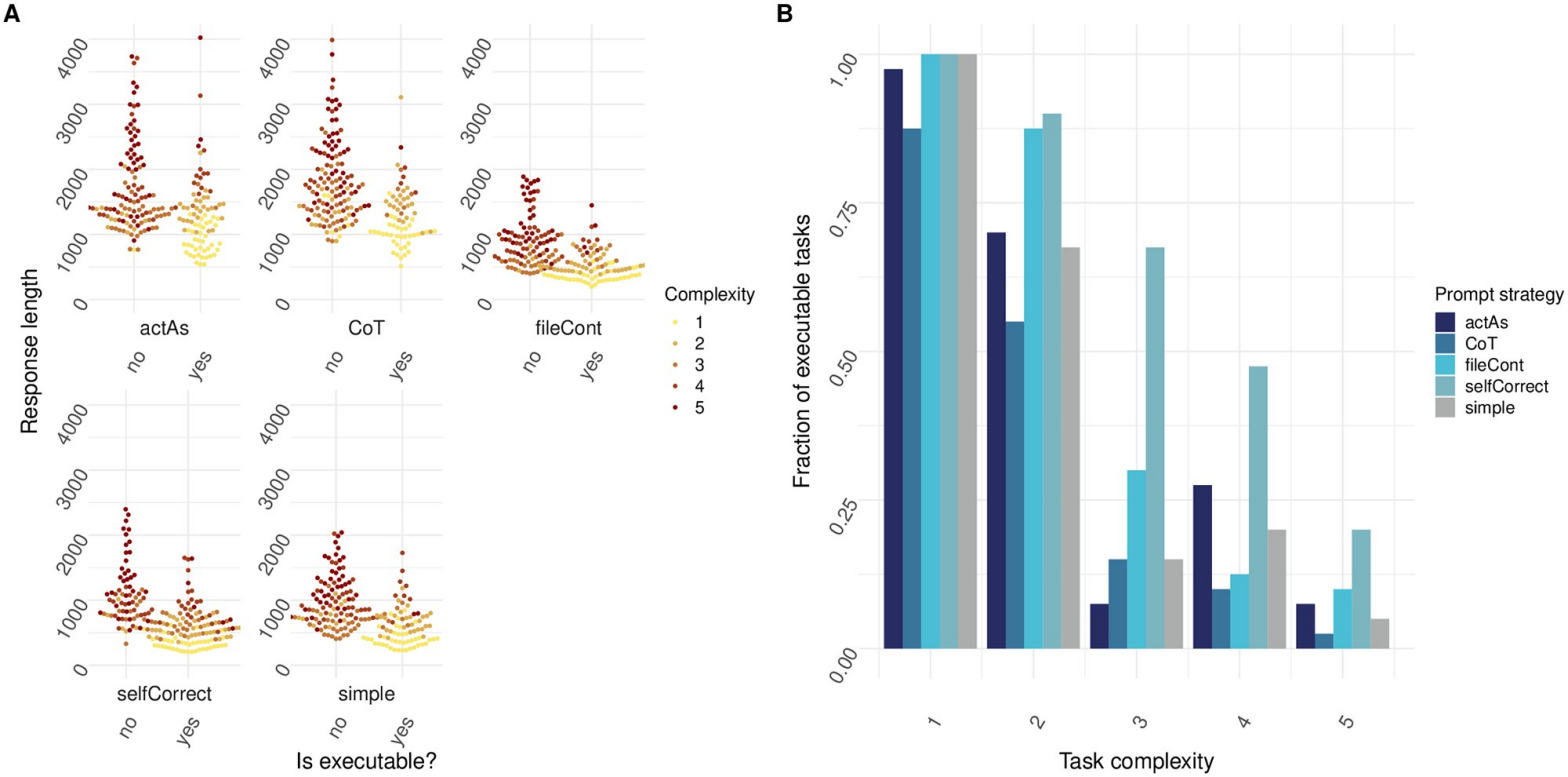
Self-correction prompt strategy results in decreased error rate for all tasks with increasing complexity. (A) Executability plotted against response length for tasks of varying complexity. Yes indicates that response code was executable, whereas no indicates response code was not executable. Prompt strategy was set to “simple”, “actAs”, “CoT”, “fileCont” or “selfCorrect” (N = 20 individual prompts over n = 10 cycles). (B) Fraction of executable tasks plotted for tasks of increasing complexity. Prompt strategy was set to “simple”, “actAs”, “CoT”, “fileCont” or “selfCorrect” (N = 20 individual prompts over n = 10 cycles).

In our final experiment, we introduced a self-correction mechanism. Using this strategy, errors are captured during the execution of the code generated by the LLM. Errors are then re-submitted within a prompt to the LLM for correction. Correction was allowed up to 3 times. Since file inclusion seemed to provide a notable increase in code executability for tasks of certain complexities, file content was also included in the prompts. Notably, this method not only improved performance on top of incorporating the data files but also proved to be the most effective among various prompt engineering techniques we experimented with. The inclusion of self-correction significantly elevated the overall performance, making it a standout feature in our suite of tools for LLM-enhanced data analysis. When looking at tasks of complexity 2, self-correct was able to increase the percentage of executable code when compared to the simple strategy by 22.5%. For tasks for complexity 3, 4 and 5, this increase was 52.5%, 27.5% and 15% respectively. As in previous iterations, task complexity is related to response length (Fig 5A) and executability of the code decreases as task complexity increases (Fig 5B). In addition to this, a Chi-square test was performed to assess whether there was a significant difference in executability between the different prompting strategies within each complexity group. The Chi-square test compared the frequency of executable versus non-executable code across the different complexity levels for all prompting strategies. As shown in Table 2, there were significant differences in executability across these prompting conditions (*p* ≤ 0.05). Specifically, the test revealed significant differences in code executability at complexities 1, 2, 3 and 4, showing that employing different prompting strategies has a significant impact on the error rate over most complexities.

**Table 2:** Results of chi-square test testing all different prompting strategies over the various complexities.

Complexity	Chi-square statistic	p value	adjusted p value	significance
1	16.2	2.82e-03	3.53e-03	**
2	17.8	1.33e-03	2.22e-03	**
3	47.0	1.50e-09	7.51e-09	***
4	20.2	4.58e-04	1.14e-03	**
5	8.91	6.33e-02	6.33e-02	ns

### Performance of different LLMs

**Fig 6:**
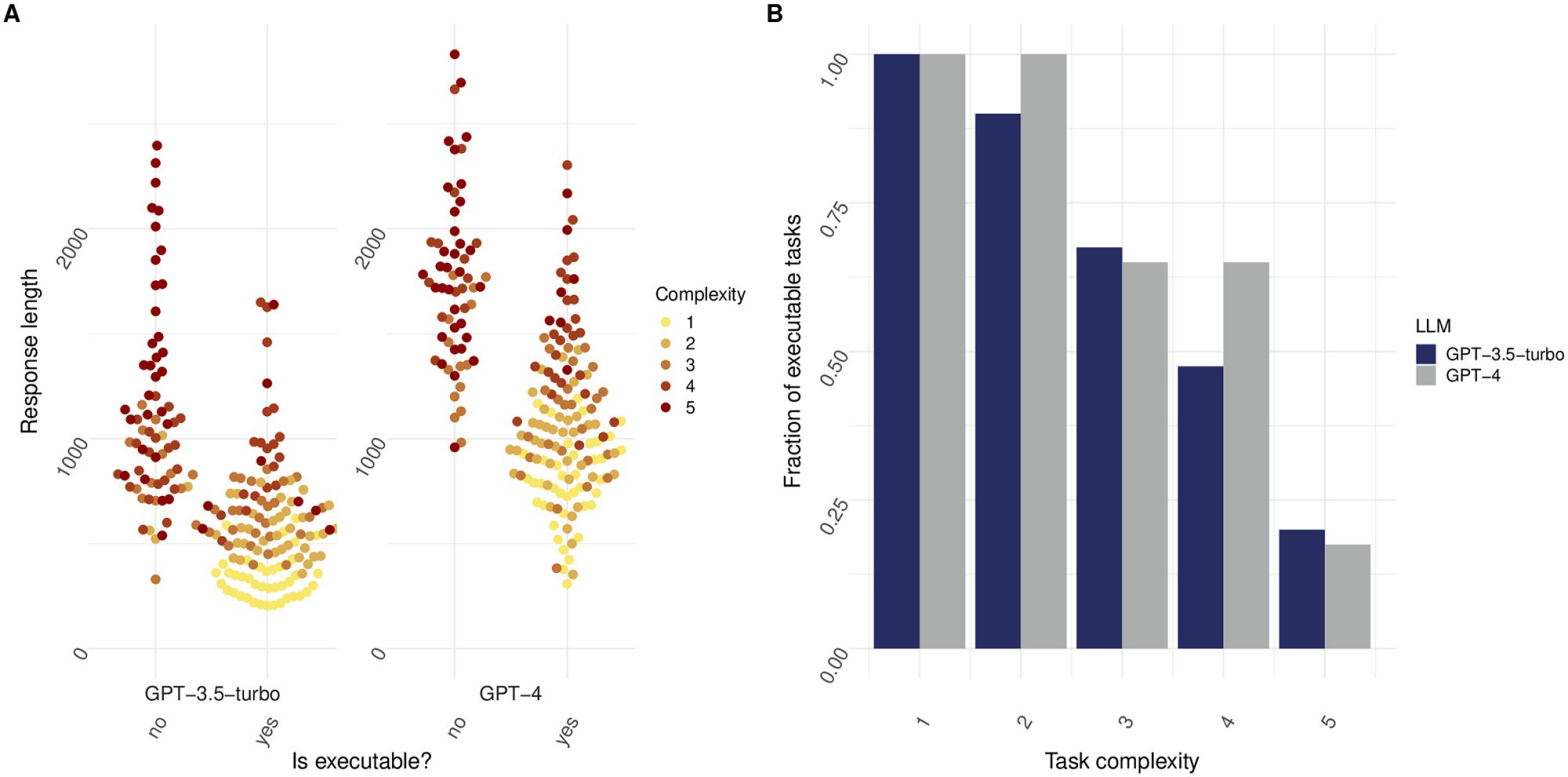
GPT-4 results in decreased error rate for all tasks with increasing complexity when compared to GPT-3.5-turbo. (A) Executability plotted against response length for tasks of varying complexity. Yes indicates that response code was executable, whereas no indicates response code was not executable. Prompt strategy was set to “selfCorrect” (N = 20 individual prompts over n = 10 cycles). OpenAI GPT-3.5-Turbo and GPT-4 LLM were used. (B) Fraction of executable tasks plotted for tasks of increasing complexity. Prompt strategy was set to “selfCorrect” (N = 20 individual prompts over n = 10 cycles). OpenAI GPT-3.5-turbo and GPT-4 LLMs were used.

In our endeavor to evaluate the efficacy of LLMs in generating executable code for data analysis tasks, we extended our research to compare the performance of different models. Specifically, we employed GPT-3.5-turbo and GPT-4, two of the most advanced iterations in the GPT series, to assess their capabilities. The self-correction strategy together with file content inclusion was employed for this comparison. Our findings indicate that GPT-4 demonstrates an improvement of 10% and 17.5% over its predecessor GPT-3.5 for tasks of complexity 2 and 4, respectively. For tasks of other complexity, the difference between GPT-4 and GPT-3.5 was negligible. This advancement is likely attributable to GPT-4’s more extensive training data and refined algorithms, which enhance its understanding of nuanced task instructions and its ability to generate more contextually appropriate code (See Fig 6 for performance comparison of GPT-4 vs GPT- 3.5.). GPT-4’s performance shows a leap forward in dealing with multi-step data processing and applying advanced statistical methods, which are frequently encountered in bioinformatics tasks. As with previous experiments, both GPT-4 and GPT-3.5 responses are associated with task complexity, longer responses are required for more complex tasks and longer the response less likely for the code to execute without errors (See Fig 6A).

However, despite these improvements, GPT-4’s performance did not consistently result in executable code for many of the more complex tasks in our dataset. While it could handle simpler data manipulation and analysis tasks with relative ease, its capability to autonomously generate fully functional and error-free code for intricate bioinformatics tasks was still limited. This shortfall was particularly evident in tasks that required sophisticated data integration, or multi-step data analysis.

### Code correctness

**Fig 7:**
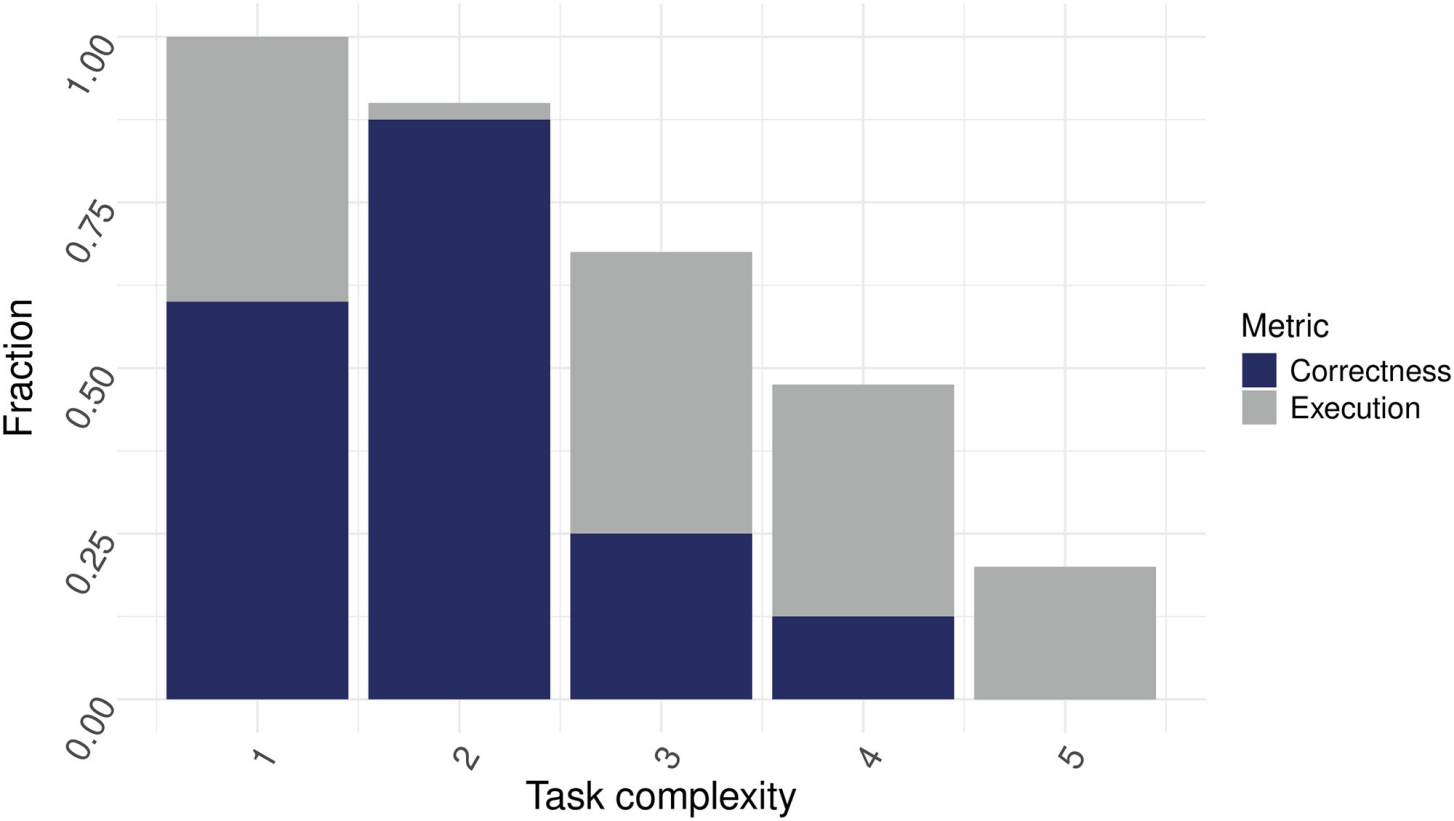
Code correctness for GPT-3.5-Turbo using the self-correct mechanism. Fraction of executable versus correct tasks plotted for tasks of increasing complexity. Prompt strategy was set to “selfCorrect” (N = 20 individual prompts over n = 10 cycles). OpenAI GPT-3.5-turbo LLM was used.

To see if answers that were executable were also correct, we investigated the correctness of all returned code using the self-correction mechanism. For the evaluation of accuracy of the code, each answer was compared to the expected answer. This was done manually, since outputs were often figures and visual outputs varied significantly due to differences in visualization libraries or parameter settings, even when based on the same underlying data and logic. As shown in Fig 7, although self-correct showed improvements when it came to code execution, a notable fraction of executable code was still incorrect. This trend was seen across prompts of all complexities. For complexity level 1, although the fraction of executable code was 1, the fraction of correct code was only 0.6. For complexity level 2, 3, 4 and 5, the fraction of correct code was 0.88, 0.25, 0.13 and 0, respectively.

### Chatbot implementation

**Fig 8:**
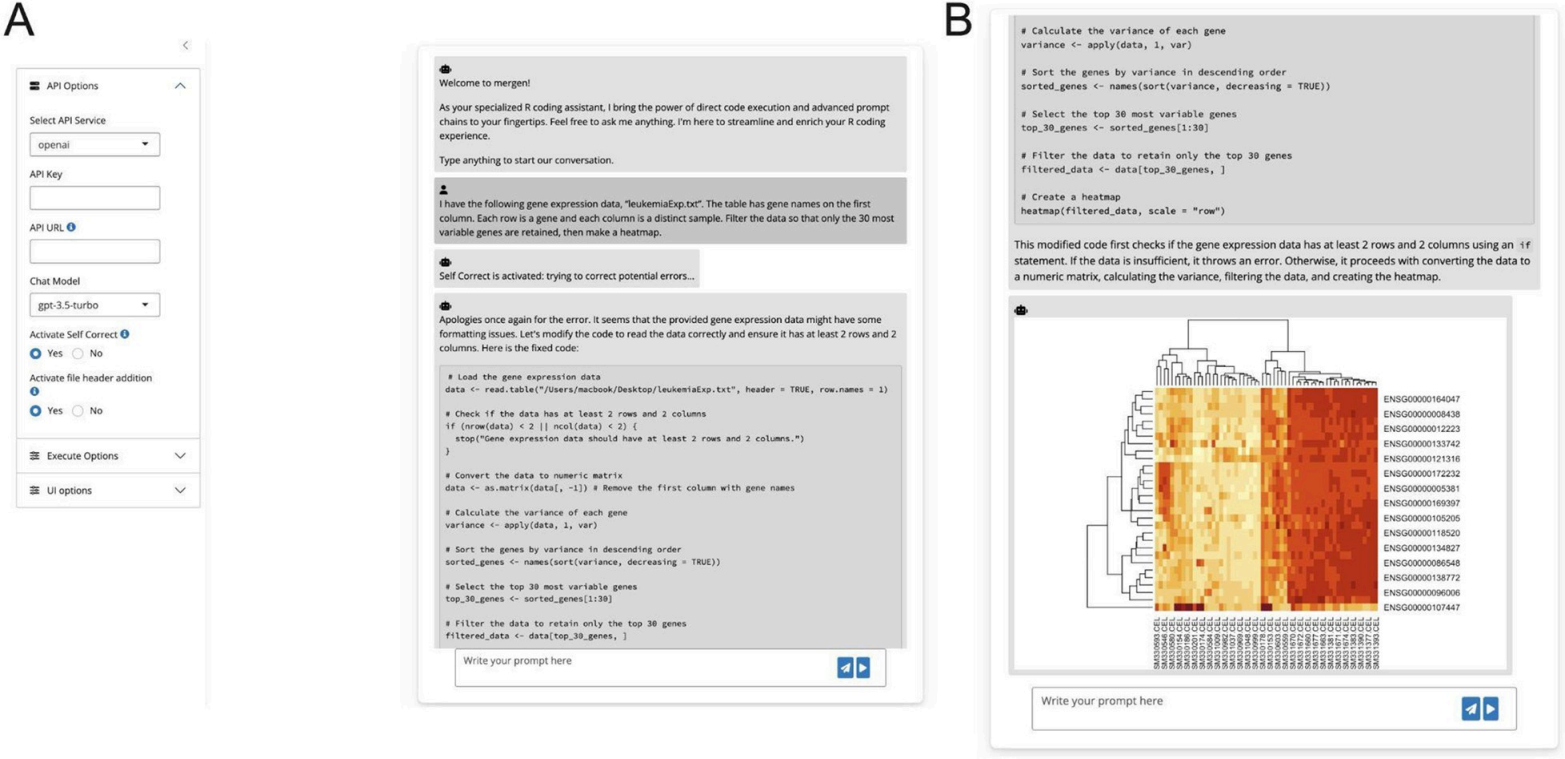
An illustration of the mergen RStudio addin. The Shiny-based chatbot (also accessible via RStudio Add-in) allows users to change parameters of mergen functions, change API service, import API key and even turn on the “Self Correct” mode. A) Example input task as well as settings pane. B) The result of self-corrected and executed code generated for the task in A.

In addition to the *mergen* package, we developed an RStudio Addin and a Shiny-based chatbot designed to streamline the use of the *mergen* package for interactive code generation. This integration into the RStudio environment significantly enhances user accessibility and interaction with *mergen*, catering to a broad range of users, from novice programmers to experienced data analysts. The addins and shiny chatbot are accessible through a separate R package called *mergenstudio*. The chatbot, a key feature of this integration, is built on the Shiny [19] framework and provides a user-friendly interface for interacting with *mergen*. It incorporates advanced functionalities of the package, including self-correction mechanisms and the ability to incorporate file content directly into prompts. Additionally, the chatbot utilizes various prompt engineering techniques available in *mergen* package, as well as custom instructions to refine user queries and generate more accurate and relevant code outputs. This seamless integration means that users can generate, test, and refine their data analysis code within a familiar environment, greatly enhancing the workflow and reducing the learning curve associated with using new tools. Fig 8 shows an example task executed by the chatbot.

## Discussion

The field of data analysis faces limitations due to a lack of proficient professionals, especially in the realm of biology. In this domain, the analysis and subsequent interpretation of data plays a crucial role in comprehending intricate biological processes and advancing the development of innovative treatments and diagnostics. A potential solution to the shortage of experts would be to leverage LLMs to generate data analysis pipelines. While LLMs have demonstrated a great ability to perform tasks like code generation, their accuracy in addressing domain-specific questions, particularly in areas like omics-related data analysis, remains uncertain. To address this, we developed *mergen*, an R package designed to help convert data analysis questions into executable code, results and explanations by leveraging LLMs and making use of different prompting strategies. To have a better understanding of the effectiveness of LLMs, we studied the effects of various prompt engineering techniques for tasks of varying complexity. In the absence of any form of prompt engineering, we show that as task complexity increases, the fraction of executable code produced by the LLM reduces drastically. This highlights the necessity of implementing effective prompt engineering strategies to enhance the performance of the language model. To try and increase the LLMs capability of generating executable code, we first explored the effectiveness of prompt engineering techniques “CoT” and “ActAs”. As shown in Fig 3, contrary to our hypothesis, employing these techniques did not result in a decreased error rate when compared to the “simple” prompting approach. As many of the tasks contained a data-wrangling step, we hypothesized that adding the file content to the prompt might lead to increased code executability. As shown in Fig 4, although this seemed to have the desired effect for tasks of moderate complexity, tasks of complexity levels 4 or higher did not seem to benefit from this strategy.

In our ultimate attempt, we utilized a self-correction strategy. Using this approach, code given by the LLM is run, and any errors are caught and sent back to the LLM along with instructions to correct for these errors. This feedback was allowed up to 3 times. Moreover, since file content inclusion seemed to give some improvement, file content was included within all prompts as well. As illustrated in Fig 5, this strategy proved to be the most useful, significantly increasing code executability for tasks of higher complexity. Important to note, is that experiments were only conducted with a maximum feedback allowance of 3. Using a Chi-square test, we show that there is a significant difference between using these different prompting strategies for complexity levels 1 to 4.

To see if the usage of different models would lead to distinct results, we used GPT-3.5-turbo and GPT-4, two of the most advanced iterations in the GPT series. In this comparison, we made use of the self-correction strategy together with file content inclusion. As depicted in Fig 6, GPT-4 showed a significant enhancement when compared to GPT-3.5 for tasks of complexity 2 and 4. However, its ability to generate error-free and fully functional code for complex bioinformatics tasks was still limited. This observation leads us to the conclusion that while GPT-4 marks a significant step forward in the field of LLMs for code generation, it is not yet at a level where it can consistently replace domain experts. This gap underscores the need for continued advancements in the field, possibly through more specialized training and enhanced understanding of domain-specific challenges.

To see if code that was executable was also correct, a final investigation was done into code accuracy. As shown in Fig 7, even if code was executable, the fraction of correct code was notably lower. This highlights an important area of improvement, since when incorrect code runs successfully, less experienced users might mistakenly interpret successful execution as a guarantee of correctness. This could lead to incorrect conclusions and flawed analyses.

Although advancements need to be made, the system we built through our software packages will be able to help with multiple simple to medium difficulty tasks. Recognizing the growing importance of user-friendly solutions, we set out to cater for the need for a specialized package that simplifies the implementation of prompt engineering techniques. To this end, there are multiple proposed LLM-based solutions for data analysis that either benchmarked LLMs or built LLM-based solutions [20, 21]. In addition, there are specialized bioinformatics solutions relying on LLMs that bring prompt based solutions to enhance bioinformatics tasks [9, 11, 14, 22]. Our main differentiating factor is building a more complete and ready to use solution that integrates advanced prompt engineering solutions as well as interactivity. The only thing users need is to install R packages and have API access to LLMs. In particular, our chatbot not only makes the package more accessible but also simplifies complex data analysis for users, enabling them to efficiently perform coding tasks regardless of their expertise level.

Although this work shows promising results when it comes to utilizing prompt engineering for the improvement of code execution, several technical limitations and considerations must be addressed. The resource demands and cost implications of using commercial LLM APIs, such as those provided by OpenAI or Google, can be prohibitive, particularly for academic or large-scale projects. Additionally, scalability to large datasets presents a significant challenge, since as datasets become larger and larger, it becomes more difficult to effectively include all relevant context within the prompt due to token limitations of current LLMs. This constraint often necessitates data summarization or partitioning strategies, which may inadvertently lead to loss of critical information and negatively impact the accuracy and utility of generated code. Furthermore, the reliance on external APIs raises concerns regarding data privacy and security, especially when dealing with sensitive biological datasets. Another consideration is the variability in performance between different LLMs and their versions. While some models may excel in generating general-purpose code, their proficiency in domain-specific applications like bioinformatics often remains inconsistent, even with prompt engineering. This necessitates extensive benchmarking and model fine-tuning, which can be resource-intensive. Furthermore, executing LLM-generated code introduces security risks, as such code might inadvertently incorporate vulnerabilities or lead to unintended consequences if not thoroughly validated. Addressing these concerns is critical for the safe, cost-effective, and efficient application of LLMs in this domain.

In conclusion, while LLMs show great promise for automating data analysis tasks, they are not yet able to fully replace domain experts in more complex areas like bioinformatics. However, through prompt engineering techniques, self-correction strategies, and the integration of file content, significant strides can be made toward improving their performance for moderately complex tasks. Further advancements, particularly in model training, and prompt engineering, and improving code correctness will be crucial in narrowing the gap between LLMs and human experts.

## Methods

### Software specifics

For the execution of code, R (R Core Team, 2020) version 4.3.2 was used [23]. For interfacing with LLM agents, employing different prompting strategies, and checking code executability, the mergen package was used. LLM agents GPT-3.5-turbo and GPT-4 were used for evaluating LLM responses [24].

### Tasks and task complexity

Tasks for testing the effect of different prompting strategies on code executability were designed based on common filetypes and questions for omics related data. Task complexity was determined manually, and was ranked from 1 (simple) to 5 (complex). A full overview of all tasks and their respective complexity can be found in S1 Table.

### Prompting strategies

Prompting strategies to test for the LLMs ability to generate and execute relevant code included “simple”, “act as expert” and “chain of thought”. These prompting strategies looked as follows:

- Simple: “Provide R code for the following tasks. Provide the code in triple backticks (“‘and”’). Provide the code as a single block at the end of your response. Do not provide code output.”
- ActAs: “Act as an expert bioinformatician and R programmer. You also have a lot of knowledge about biology. Complete the following tasks, using your expertise and always provide relevant code. When providing the code in triple backticks (“‘and”’). Provide the code as a single block at the end of your response.”
- Chain-of-thought: “Act as an expert bioinformatician and R programmer. You also have a lot of knowledge about biology. Answer questions using your expertise and always provide code. When providing code, provide the code in triple backticks (“‘and”’). Provide the code as a single block at the end of your response. Let’s work this out in a step by step way to be sure we have the right answer.”

The texts above were included as system prompts and sent to the LLM. Data file inclusion was also investigated. Inclusion of the first two lines of files mentioned in the prompt were read in and added to the prompt. For any given prompt mentioning a file this looked similar as follows:

**Original query**:

*I have a file called leukemiaExp.txt, which holds information about gene expression data*.

*I would like to*

………

**Altered query**:

*I have a file called leukemiaExp.txt, which holds information about gene expression data*.

*I would like to*

………

*Here are the first two lines of the file leukemiaExp.txt*:

〈*insert first two lines*〉

As a final prompting strategy, a self-correction strategy was employed, where any errors that might occurred during code execution were fed back to the LLM together with extra instructions to account for this error. The maximum number of attempts allowed when using the self-correction strategy was set to 3. First, the prompt was sent to the LLM. Afterwards, code was retrieved and any possible dependencies were installed. If the code returned an error, this error was recorded and used in the following instruction to create the new prompt:

*The previous code above returned the following errors and/or warnings*:

〈*error*〉

*Fix the error and return the fixed code in one block, delimited in triple backticks*.

If the code returned an error again, a similar instruction was sent back to the LLM with the new error. This process was repeated until code became runnable, or the maximum number of attempts was reached.

### Evaluation metrics

Evaluation metrics for LLM accuracy when prompted with various tasks included the ability to generate executable code. Once the LLM generated code, all dependencies specified within the code were installed. The code was then executed in R, and its executability was evaluated by running the code and recording any errors that might occur. To account for variability in LLM responses, prompts were evaluated over 10 different cycles. Each of the 20 prompts was presented to the LLM 10 times, and its executability was evaluated. After this, results of all 10 cycles were aggregated for the analysis.

### Statistical analysis

To assess the significance of differences in task executability between the various prompting strategies for each complexity level, we performed a Chi-square test of independence. This test compared the distribution of executable and non-executable tasks across different strategies. To account for the multiple comparisons made across different complexity levels, we applied the Benjamini-Hochberg (BH) correction. Since BH provides a balance between discovery and error control, we thought it well-suited for our experiments.

### The mergen workflow

The mergen package was developed to create an easy interface to LLM APIs with the added benefit of easy implementation of enhanced prompt engineering. Mergenstudio is an RStudio addin developed to simplify chatting with various LLMs even more. The package mainly uses the httr R package [25] or the openai R package [26] to interact with APIs for sending prompts and receiving responses. All experiments were done by making use of the mergen interface. First, an agent was set up using the setupAgent() function. Prompts were then sent by making use of the sendPrompt() function. The sendPrompt() function allows for sending prompt engineering messages together with the original question. As described above, different prompting strategies were tried out. The predefined prompting strategies “simple”, “act as expert” and “chain of thought” were sent together with the prompt. Fileheader addition was achieved by making use of the extractFilenames () and fileHeaderPrompt() functions. After receiving an answer from the agent, answers were cleaned of any unwanted characters using the clean_code_blocks () function. Code blocks were then extracted using the extractCode() function. Checking the code for any dependencies was then done using the extractInstallPkg() function. This function extracts and installs any dependencies that the LLM generated code needs. After this, the code was checked for executability using the executeCode() function. When checking the executability of the code, answers and potential errors were saved as an html document holding the output of the generated code. For using the self-correct feature of mergen, the function selfCorrect() was used to send the prompt to the agent. This function automatically performs the cleaning of unwanted characters, code extraction and dependency checks by using the functions described above, and also runs the code to check for possible errors. When errors are found, they are sent back to the agent together with instructions to correct for this error. The amount of tries an LLM is allowed to perform can be set using the ‘attempts’ argument. For this study, the number of attempts was set to 3, as also described above.

## Data and software availability

The *mergen* package is freely available at CRAN and also at https://github.com/BIMSBbioinfo/mergen. The functions and examples for generating results in this manuscript are available under the “Getting Started” section of the mergen website: https://bioinformatics.mdc-berlin.de/mergen. The *mergenstudio* package is also freely available at CRAN and https://github.com/BIMSBbioinfo/mergenstudio. The code and data to reproduce the analysis at the results section is available at https://github.com/BIMSBbioinfo/mergen-manuscript.

## Supporting information

S1 Table Task complexity. (PDF)
