# Supplementary material for: Leveraging large language models for data analysis automation
Source: PLoS One. 2025 Feb 21;20(2):e0317084. doi: 10.1371/journal.pone.0317084 (PMC11844886; doi:10.1371/journal.pone.0317084)
Supplement: S1 Table — (PDF) [file pone.0317084.s001.pdf]

Supplementary Tables

Table S1. Task complexity and prompts used for evaluation

Task complexity	Prompt
1	I have a tab separated file called “subjects.txt”. Read it and tell me how many rows and columns it has.
1	I have the following gene expression data, “leukemiaExp.txt”. Each row is a gene and each column is a distinct sample. The file is tab separated. The table has gene names on the first column. How many genes and samples does the dataset have?
1	I have the following gene expression data as an excel sheet, “Supplementary Table 1 exp.xlsx”. Each row is a gene and each column is a distinct sample. First column contains gene names. How many genes and samples does the dataset have?
1	I have the following count table for an RNA-seq exper- iment,“SRP021193.raw counts.tsv”. The first column contains the gene names, the last column is gene length and the rest are samples.The file is tab separated. How many genes and samples does the dataset have?
2	I have the following gene expression data, “leukemia- Exp.txt”.The file is tab separated. The table has gene names on the first column. Each row is a gene and each column is a distinct sample. Filter genes based on their variability so we retain the most variable top 1000 genes.
2	I have the following gene expression data, “leukemi- aExp.txt”. The file is tab separated. The table has gene names on the first column. Each row is a gene and each column is a distinct sample. If there are more samples than 500 then randomly sample the samples column and create a subset of the data
2	We have the following datasets. One of the dataset contains CpG

	methylation values per CpG and per individual as a table this is contained in the “metRmOlWithDbgapIdHeader.txt” file. Each column is an individual represented by a DBGapId, which is in- cluded as the first row. Each row is a CpG represented by “CpG id”, values in the table are methylation values. Another data set is contained in “subjects.txt”. This file has the information on the individuals. Most important feature for us here is the “Age” column. The “dbGap ID” column (second column) in this table should match the first row of the “metRmOlWithDb- gapIdHeader.txt”, and they represent the same indi- viduals. Read the tables count number of rows and columns for each.
2	I have the following count table for an RNA-seq experiment, rows are genes and columns are sam- ples,“Supplementary Table 1 exp.xlsx”. The first col- umn of the file is my gene names and the rest of the columns are samples. My annotation data is here “SRP021193.colData.tsv”, it contains sample annota- tions and rows of this matches the columns of count table, first column is the sample ids. Read the tables count number of rows and columns for each.
3	I have the following count table for an RNA-seq experiment, rows are genes and columns are sam- ples,“Supplementary Table 1 exp.xlsx”. The first col- umn of the file is my gene names and the rest of the columns are samples. My annotation data is here<br><br>“SRP021193.colData.tsv”, it contains sample annota- tions and rows of this matches the columns of count table, first column is the sample ids. Merge annotation data and gene expression tables and calculate number of columns.
3	We have the following datasets. One of the dataset contains CpG methylation values per CpG and per individual as a table this is contained in the “metR- mOlWithDbgapIdHeader.txt” file. Each column is an individual represented by a DBGapId, which is in- cluded as the first row. Each row is a CpG represented by “CpG id”, values in the table are methylation val- ues. Another data set is contained in “subjects.txt”. This file has the information on the individuals. Most important feature for us here is the “Age” column. The “dbGap ID” column (second column) in this table should match the first row of the “metRmOlWithDb- gapIdHeader.txt”, and they represent the same indi- viduals. Read the data and merge them into a single table using dbGapId information, return the number of columns.

3	I have the following gene expression data, “leukemi- aExp.txt”. The file is tab separated. The table has gene names on the first column. Each row is a gene and each column is a distinct sample. If there are more samples than 500 then randomly sample the samples column and create a subset of the data. Create a boxplot of the columns
3	I have the following gene expression data, “leukemi- aExp.txt”. The file is tab separated. The table has gene names on the first column. Each row is a gene and each column is a distinct sample. Filter genes based on their variability so we retain the most vari- able top 1000 genes. Create a scatterplot of the two most variable genes.
4	I have the following gene expression data, “leukemi- aExp.txt”. The file is tab separated. The table has gene names on the first column. Each row is a gene and each column is a distinct sample. Filter genes based on their variability so we retain the most vari- able top 1000 genes. Based on these variable genes, plot a heatmap with clustering. Also plot PCA for samples.
4	I have the following gene expression data, “leukemiaExp.txt”. The file is tab separated. The table has gene names on the first column. Each row is a gene and each column is a distinct sample. Filter genes based on their variability so we retain the most variable top 1000 genes. Based on these variable genes cluster the samples, and extract cluster specific genes for each cluster.
4	We have the following datasets. One of the dataset contains CpG methylation values per CpG and per individual as a table this is contained in the “metR- mOlWithDbgapIdHeader.txt” file. Each column is an individual represented by a DBGapId, which is in- cluded as the first row. Each row is a CpG represented by “CpG id”, values in the table are methylation val- ues. Another data set is contained in “subjects.txt”. This file has the information on the individuals. Most important feature for us here is the “Age” column. The “dbGap ID” column (second column) in this table should match the first row of the “metRmOlWithDb- gapIdHeader.txt”, and they represent the same indi- viduals. Read the data and merge them into a single table using dbGapId information. Plot

	a scatter plot of CpG methylation values for the two oldest subjects in the samples.
4	I have the following count table for an RNA-seq experiment, rows are genes and columns are sam- ples,“Supplementary Table 1 exp.xlsx”. The first col- umn of the file is my gene names and the rest of the columns are samples. My annotation data is here<br><br>“SRP021193.colData.tsv”, it contains sample annota- tions and rows of this matches the columns of count table, first column is the sample ids. Merge annota- tion data and gene expression tables. Plot boxplots for gene expression values per sample and color code boxplots based on group” variable in my annotation data.
5	We have the following datasets. One of the dataset contains CpG methylation values per CpG and per individual as a table this is contained in the “metR- mOlWithDbgapIdHeader.txt” file. Each column is an individual represented by a DBGapId, which is in- cluded as the first row. Each row is a CpG represented by “CpG id”, values in the table are methylation val- ues. Another data set is contained in “subjects.txt”. This file has the information on the individuals. Most important feature for us here is the “Age” column. The “dbGap ID” column (second column) in this table should match the first row of the “metRmOlWithD- bgapIdHeader.txt”, and they represent the same in- dividuals. Build a predictive model to predict Age from methylation values, and display most important variables for the predictive models.
5	We have the following datasets. One of the dataset contains CpG methylation values per CpG and per individual as a table this is contained in the “metR- mOlWithDbgapIdHeader.txt” file. Each column is an individual represented by a DBGapId, which is in- cluded as the first row. Each row is a CpG represented by “CpG id”, values in the table are methylation val- ues. Another data set is contained in “subjects.txt”. This file has the information on the individuals. Most important feature for us here are the “Age”,“sex” and “Race” columns. The “dbGap ID” column (second column) in this table should match the first row of the “metRmOlWithDbgapId.txt”, and they represent the same individuals. Find all CpGs associated with Age but not with sex or Race, and display top 20 CpGs.

5	I have the following count table for an RNA-seq experiment, rows are genes and columns are samples,“Supplementary Table 1 exp.xlsx”. The first column of the file is my gene names and the rest of the columns are samples. My annotation data is here<br><br>“SRP021193.colData.tsv”, it contains sample annota- tions and rows of this matches the columns of count table, first column is the sample ids. Normalize counts, make two PCA plots for samples, one color coded samples by “group” and the other color coded by “Li- brarySelection” in my annotation data.
5	I have the following count table for an RNA-seq experiment, rows are genes and columns are sam- ples,“Supplementary Table 1 exp.xlsx”. The first col- umn of the file is my gene names and the rest of the columns are samples. My annotation data is here “SRP021193.colData.tsv”, it contains sample annota- tions and rows of this matches the columns of count table, first column is the sample ids. Find genes spe- cific for each “group” in my annotation data using statistical tests. Plot PCA only using “group” specific genes and color code PCA by “group” variable in my annotation data.
